# Evaluating Sensory Acuity as a Marker of Balance Dysfunction After a Traumatic Brain Injury: A Psychophysical Approach

**DOI:** 10.3389/fnins.2020.00836

**Published:** 2020-08-11

**Authors:** Rakesh Pilkar, Kiran K. Karunakaran, Akhila Veerubhotla, Naphtaly Ehrenberg, Oluwaseun Ibironke, Karen J. Nolan

**Affiliations:** ^1^Center for Mobility and Rehabilitation Engineering Research, Kessler Foundation, West Orange, NJ, United States; ^2^Department of Physical Medicine and Rehabilitation, Rutgers New Jersey Medical School, Newark, NJ, United States; ^3^New Jersey Institute of Technology, Newark, NJ, United States; ^4^Children’s Specialized Hospital, New Brunswick, NJ, United States

**Keywords:** sensory threshold detection, posturography, traumatic brain injury, balance, rehabilitation

## Abstract

There is limited research on sensory acuity i.e., ability to perceive external perturbations via body-sway during standing in individuals with a traumatic brain injury (TBI). It is unclear whether sensory acuity diminishes after a TBI and if it is a contributing factor to balance dysfunction. The objective of this investigation is to first objectively quantify the sensory acuity in terms of perturbation perception threshold (PPT) and determine if it is related to functional outcomes of static and dynamic balance. Ten individuals with chronic TBI and 11 age-matched healthy controls (HC) performed PPT assessments at 0.33, 0.5, and 1 Hz horizontal perturbations to the base of support in the anterior-posterior direction, and a battery of functional assessments of static and dynamic balance and mobility [Berg balance scale (BBS), timed-up and go (TUG) and 5-m (5MWT) and 10-m walk test (10MWT)]. A psychophysical approach based on Single Interval Adjustment Matrix Protocol (SIAM), i.e., a *yes-no* task, was used to quantify the multi-sensory thresholds of perceived external perturbations to calculate PPT. A mixed-design analysis of variance (ANOVA) and *post-hoc* analyses were performed using independent and paired t-tests to evaluate within and between-group differences. Pearson correlation was computed to determine the relationship between the PPT and functional measures. The PPT values were significantly higher for the TBI group (0.33 Hz: 2.97 ± 1.0, 0.5 Hz: 2.39 ± 0.7, 1 Hz: 1.22 ± 0.4) compared to the HC group (0.33 Hz: 1.03 ± 0.6, 0.5 Hz: 0.89 ± 0.4, 1 Hz: 0.42 ± 0.2) for all three perturbation frequencies (*p* < 0.006 post Bonferroni correction). For the TBI group, the PPT for 1 Hz perturbations showed significant correlation with the functional measures of balance (BBS: *r* = −0.66, *p* = 0.037; TUG: *r* = 0.78, *p* = 0.008; 5MWT: *r* = 0.67, *p* = 0.034, 10MWT: *r* = 0.76, *p* = 0.012). These findings demonstrate that individuals with TBI have diminished sensory acuity during standing which may be linked to impaired balance function after TBI.

## Introduction

Balance control is regulated within the central nervous system by the complex integration of visual, vestibular, and somatosensory pathways and motor control (Hillier et al., 1997; Greenwald et al., 2001). Traumatic brain injury (TBI) often damages the areas of the brain that regulate balance (Allison, 1999). Although the peripheral system may or may not be impaired as a secondary consequence of the same event, damage to the brain could result in impaired central motor processes such as intention to act, motor planning, and automatic postural response mechanisms (Allison, 1999). Further, impaired sensory integration, a central process, is postulated as one of the sources for imbalance after TBI (Sosnoff et al., 2011; Fino et al., 2017; Peterka et al., 2018). The body-position awareness, i.e., the detection of body-sway, is a fundamental necessity to maintain static and dynamic balance during activities of daily living (Fitzpatrick and McCloskey, 1994) and it is achieved by an accurate perception of the body’s interaction with the surrounding environment. TBI can impair the integration of the visual, vestibular, and somatosensory (proprioceptive) inputs (Allison, 1999; Sarno et al., 2003) that permits body position awareness with respect to self and the environment. Therefore, impairments to sensory pathways (Fino et al., 2017) and their integration (Peterka et al., 2018) to facilitate perception of body-environment interaction can lead to poor understanding of the surroundings, impaired balance and a greater risk of falls after TBI. Falls occur when the center of mass (CoM) is displaced beyond the base of support and when the central nervous system fails to “detect and correct” this displacement in time (Institute of Medicine (US), and Division of Health Promotion and Disease Prevention, 1992). Therefore, accurate perception is even more critical in a dynamic setting which demands attention, adaptation to external stimuli and adequate reactive motor responses for achieving balance control and avoiding falls. Further, in the domain of perception and balance, sensory acuity, i.e., the ability to detect body-sway during external perturbations (Fitzpatrick and McCloskey, 1994; Richerson et al., 2003), could stem from impaired sensory integration. Limited research specifically reports objective quantification of impairments to sensory integration after TBI (Peterka et al., 2018) and no research thus far has investigated sensory acuity in the individuals with TBI. An objective assessment of the sensory acuity, i.e., the ability to perceive external perturbations, is necessary to accurately detect, quantify, and treat sensory integration deficits that could lead to poor detection of body sway and imbalance in dynamic environment. Additionally, the outcome measure of sensory acuity can serve as a novel marker of balance function which goes beyond biomechanical and functional outcomes and may provide added information to develop rehabilitation programs aimed at improving balance and reducing falls in individuals with TBI.

Psychophysics provides a way to evaluate and quantify an individual’s sensory acuity to external stimuli (Han et al., 2016). In the realm of standing balance, psychophysical studies related to the perception of whole-body perturbations are commonly used to measure sensory acuity in terms of detection thresholds (Fitzpatrick and McCloskey, 1994; Richerson et al., 2003; Pilkar, 2011; Puntkattalee et al., 2016). Most of the research on assessing balance deficits after a TBI is restricted to biomechanical [CoM, center of pressure (CoP)] and functional outcome measures (Lehmann et al., 1990; Kaufman et al., 2006) and no research has reported psychophysical outcomes such as detection thresholds in individuals with TBI. The detection threshold quantifies the level of the external perturbation (magnitude, frequency, velocity, direction) below which the perception of the perturbation becomes unlikely (Pilkar, 2011; Pilkar et al., 2016).

The purpose of this investigation is to objectively evaluate and quantify the multi-sensory acuity to external mechanical perturbations to the base of support during standing for individuals with TBI. This multi-sensory acuity will be quantified in terms of perturbation perception threshold (PPT) using a psychophysical approach when visual, vestibular and somatosensory systems are available. The secondary objective is to determine if our novel outcome measure, PPT, is related to the functional outcomes of static and dynamic balance. Our central hypothesis is that the balance dysfunction will be characterized by an impaired PPT in addition to deficits in functional outcomes after a TBI. More specifically, individuals with TBI will exhibit elevated PPTs compared to healthy controls when experiencing external perturbations. Our secondary hypothesis is that the PPT will be correlated to the functional outcome measures, as the diminished ability to perceive changes in the body position will affect the ability of individuals with TBI to perform static and dynamic balance tasks.

## Materials and Methods

### Participants

Eleven age-matched healthy controls (HC) with no neurological, orthopedic, or visual impairments and 10 individuals diagnosed with a TBI were recruited (see Table 1). The Kessler Foundation Institutional Review Board approved all procedures and informed consent was obtained prior to study participation. Inclusion criteria for the TBI group were: (1) age between 18 and 60; (2) diagnosed with a non-penetrating TBI (≥6 months); (3) ability to stand unsupported for at least 5 min; (4) willing and able to give informed consent. Exclusion criteria for the TBI group were: (1) history of injury to the lower limbs in the past 90 days; (2) cardiac disease; (3) a previous history of balance impairments prior to TBI.

**TABLE 1:** Demographics for the study participants with data reported in terms of mean ± standard deviations.

Groups	Age (years)	Sex	Height (cm)	Weight (kg)	BMI	TSI (years)	TBI severity
HC (*n* **=** 11)	52.3 ± 5.9	6 M, 5 F	169.7 ± 7.9	87 ± 18.3	37.3 ± 27.7	–	–
TBI (*n* **=** 10)	55.6 ± 3.9	7 M, 3 F	172.2 ± 10.1	93.1 ± 25	31.1 ± 6.4	9.9 ± 17	5 mild, 2 moderate, 3 severe

*BMI, Body Mass Index; TSI, Time since injury at the time of testing. TBI severity was diagnosed at the time of injury.*

### Procedures

#### Clinical Assessments of Static and Dynamic Balance Function

Participants from both the HC and TBI groups completed clinical assessments of static and dynamic balance function including: the Berg Balance Scale (BBS); the Timed-up and Go (TUG); 5-m walk test (5MWT); and 10-m walk test (10MWT). The BBS is a 14-item assessment scale that quantitatively assesses balance during static and dynamic functional movements in adults. Each item is scored from 0 to 4, with a score of 0 representing the inability to complete the task and a score of 4 representing independent completion of the task. The maximum possible score is 56 points. The 5MWT and 10MWT are assessments of how quickly and safely an individual traverses standard distances, and the TUG evaluates a participant’s ability to transition from sitting to brief locomotor tasks and then return to a seated position.

#### PPT Assessments

**FIGURE 1:**
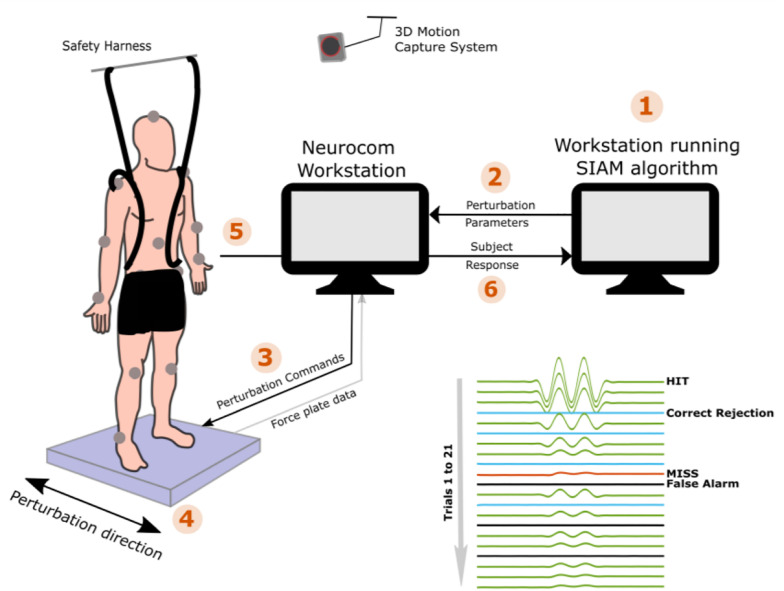
The experimental set up and procedures are demonstrated for the PPT assessments of 0.5 Hz perturbations. (1) the assessment starts with the default perturbation amplitude of 4 mm, (2) perturbation amplitude is fed to the Neurocom computer which (3) sends out the command to the on-board controller for execution of the platform movement, (4) platform moves precisely at the desired amplitudes in the anterior-posterior direction, (5) the subject reports if he/she felt the platform movement, and (6) based on the correctness of subject’s response, SIAM algorithm computes the next perturbation amplitude and the steps 1–6 are repeated for the remaining 20 trials.

**FIGURE 2:**
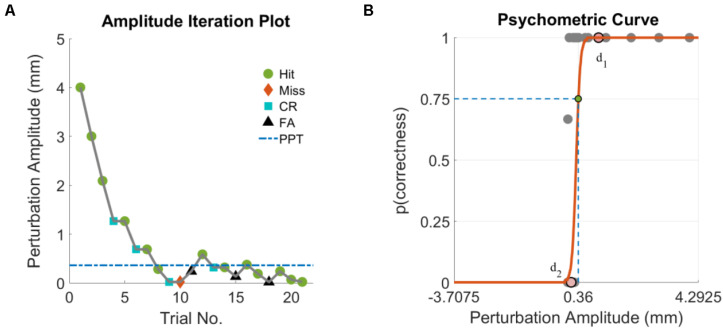
**(A)** Perturbation amplitude iterations based on a representative subject’s response for a set of PPT assessments and **(B)** corresponding psychometric curve with PPT shown by the green circle. Points d_1_ and d_2_ represent the median of perturbation amplitudes that were successfully detected (*p* = 1) and not detected (*p* = 0), respectively. HIT, perturbation presented and correctly reported; MISS, perturbation presented but not reported; Correct Rejection (CR), perturbation not presented and not reported; False Alarm (FA), perturbation not presented but reported.

PPT assessments for the HC and TBI groups were completed after completing clinical assessments of static and dynamic balance function. The NeuroCom Smart Equitest Clinical Research System (CRS) (Natus Medical Inc., Pleasanton, CA), was used to provide precise perturbations to the base of support in anterior-posterior (AP) direction (Figure 1). Perturbations were applied to the base of support at three different frequencies- 0.3, 0.5, and 1 Hz, which were selected in order to keep the perturbations within the range of natural healthy sway (<2 Hz) (Soames and Atha, 1982). For each perturbation frequency, a total of 21 trials consisting of a randomized configuration of 14 perturbation trials and 7 non-perturbation trials (2:1) were performed. Each trial lasted 15-s which included 5 s of quiet standing (QS), followed by sinusoidal translations of the platform in the AP direction at the selected perturbation frequency and programmed amplitude for 5 s (or no movement for a non-perturbation trial), followed by 5 more seconds of QS (Figure 1). At the end of each trial, the participant was verbally asked if they felt the platform move. Depending on the correctness of their *yes* or *no* response (HIT: correctly detected perturbation, MISS: non-detected perturbation, Correct Rejection: correctly reported no perturbation and False Alarm: perturbation reported for a non-perturbation trial), the amplitude of the next trial was adjusted using the Single Interval Adjustment Matrix (SIAM) algorithm with parameter estimation by sequential testing (PEST) (Taylor, 1967; Kaernbach, 1990; Pilkar, 2011; Pilkar et al., 2016). The process is shown in Figure 1 using the numbered sequences from 1 to 6. The PPT value for each frequency was computed using the psychometric curve (Algom, 1992; Puntkattalee et al., 2016) by plotting the percentage of accuracy (HIT, correct rejections) as a function of perturbation amplitude (Figure 2). A sigmoid function was used to fit the data for each frequency, and the perturbation amplitude (x-axis) where the curve achieves a 75% probability of correct detection (y-axis) was chosen as the PPT value (Figure 2B; Algom, 1992; Puntkattalee et al., 2016). This procedure was performed for all three frequencies for each participant. To familiarize the participants with perturbations and minimize the learning effect, five perturbation trials at suprathreshold amplitudes (≥4 mm peak-to-peak) were performed at each of the perturbation frequencies before the PPT assessments. No verbal response was recorded during these trials.

### Statistical Analyses

The normality of the PPT outcome was evaluated using Shapiro-Wilk test of normality. It was found that the assumption of normality was valid for the PPT data for both groups for 0.33 (HC: *p* = 0.06; TBI: 0.21) and 1 Hz (HC: *p* = 0.56; TBI: 0.2) perturbations. For 0.5 Hz perturbations, PPT data was normally distributed for the HC group (*p* = 0.1). The TBI group showed approximately normal distribution (*p* = 0.01) which was also supported by the Q-Q plots showing approximately linear data fit. Hence, the PPT data were analyzed using a mixed-design Analysis of Variance (ANOVA) with a within-subjects factor of perturbation frequency (0.33, 0.5, and 1 Hz) and a between-subject factor of condition (healthy, TBI). Mauchly’s test for sphericity indicated that the assumption of sphericity was valid [χ^2^(2) = 1.36, *p* = 0.51] for the PPT measure, hence sphericity was assumed.

For the ANOVA tests, the significance level was set to 0.05. Based on the significance of the main effects, *post-hoc* tests were performed to compute a between-subjects comparison and a within-subject comparison. A Bonferroni correction was applied to avoid type-I errors and the new significance level was corrected to 0.006. The functional outcome measures of static and dynamic balance (BBS, TUG, 5MWT, 10MWT) were compared using independent sample t-tests. In addition, the functional outcome measures were correlated with PPT using a Pearson product-moment correlation (p ≤ 0.05). The results are reported in terms of mean ± standard deviations (sd) including the PPT outcome reported in Table 2 and the functional outcomes reported in Table 3.

**TABLE 2:** Results of *post-hoc* analysis for between-group and within-group comparison of the PPT outcome (mean ± sd).

	Perturbation frequency (Hz)			Within-group
Groups	0.33	0.5	1	
Healthy (*n* **=** 11)	1.03 ± 0.6^*a,c*^	0.89 ± 0.4^*a,b*^	0.42 ± 0.2^*b,c*^	^*a*^*t*(10) = 1.1, *p* = 0.298
				^*b*^*t*(10) = 4.14, *p* = 0.002*
				^*c*^*t*(10) = 3.89, *p* = 0.003*
TBI (*n* **=** 10)	2.97 ± 1.0^ a,c^	2.39 ± 0.7^ a,b^	1.22 ± 0.4^ b,c^	^*a*^*t*(9) = 2.3, *p* = 0.047
				^*c*^*t*(9) = 7.05, *p* < 0.006*
				^*b*^*t*(9) = 5.79, *p* < 0.006*
Between-group	*t*(19) = −5.56, *p* < 0.006	*t*(19) = −6.01, *p* < 0.006	*t*(19) = −5.69, *p* < 0.006	

*p < 0.006 where 0.006 is the significance level adjusted after Bonferroni correction. ^a^0.33 vs. 0.5 Hz. ^b^0.5 vs. 1 Hz. ^c^0.33 vs. 1 Hz. Bold values represent statistically significant differences.

**TABLE 3:** Between-group comparison of the functional outcome measures (mean ± sd) of static and dynamic balance.

Groups	BBS	5MWT (s)	10MWT (s)	TUG (s)
HC (*n* **=** 11)	55.91 ± 0.3	2.96 ± 0.58	5.47 ± 0.91	7.41 ± 1.38
TBI (*n* **=** 10)	48.8 ± 6.43	4.61 ± 1.18	9.34 ± 2.6	12.7 ± 3.6
Between-group	*t*(10) = 3.68, ***p* = 0.007**	*t*(10) = −4.15, ***p* = 0.001**	*t*(10) = −4.64, ***p* < 0.005**	*t*(10) = −4.52, ***p* < 0.005**

*Bold values represent statistically significant differences.*

## Results

### Perception of Perturbation Threshold (PPT)

**FIGURE 3:**
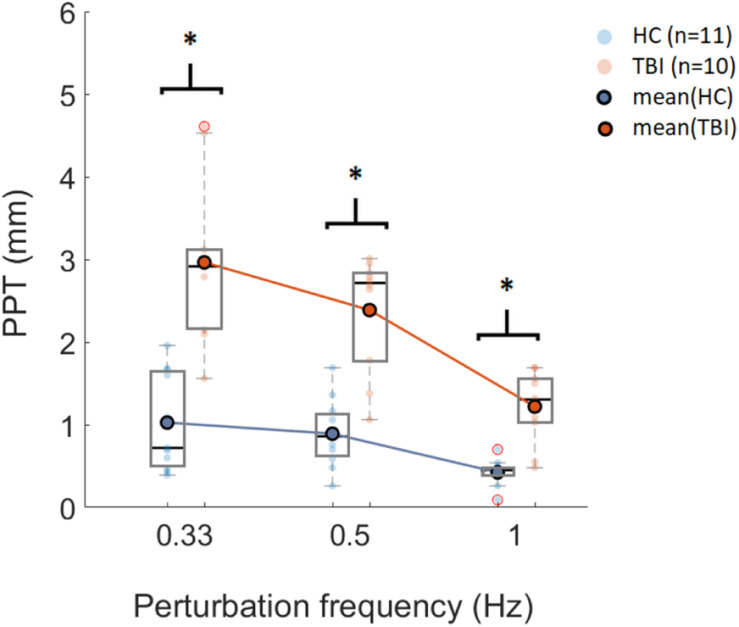
Box plot representation of PPT values computed using SIAM for three sets of perturbation frequencies (x-axis) for HC (*n* = 11) and TBI (*n* = 10). Horizontal lines in each box represent the median values. Data points shown with red circles are the outliers. ^∗^*p* < 0.006 (significance level post Bonferroni correction).

The SIAM algorithm successfully converged to the threshold amplitudes for each participant in both groups. The PPTs computed using a classical psychometric approach showed a decreasing trend with increasing perturbation frequency for both groups (Figure 3). For both groups, PPTs computed for 0.33 Hz showed the highest variability while 1 Hz perturbations showed the lowest variability based on the standard deviations. A mixed-design ANOVA showed significant main effects of perturbation frequency [*F*(2, 38) = 42.14. *p* < 0.005], and condition [*F*(1, 19) = 44.35, *p* < 0.005] on PPT, and interactions between perturbation frequency and condition [*F*(2, 38) = 9.65, *p* < 0.005].

*Post-hoc* analysis showed that there was a significant difference in PPT values between the HC group and the TBI group for all three frequency sets (*p* < 0.006) (see Table 2). Furthermore, *post-hoc* analysis showed no significant difference between frequencies of 0.33 Hz and 0.5 Hz for the within-group comparison for the HC group (*p* = 0.298) and the TBI group (*p* = 0.047). The PPT values obtained for 0.5 Hz were significantly different than those obtained for 1 Hz for both the HC group (*p* = 0.002) and the TBI group (*p* < 0.006) (Table 2). Similarly, the PPT values for 0.33 Hz were significantly higher than 1 Hz for both groups (HC: *p* = 0.003; TBI: *p* < 0.006) (Table 2).

### Correlation Between the Functional Outcomes and the PPT

The TBI group showed significantly lower scores on functional assessments compared to the HC group (Table 3). The BBS was significantly lower for the TBI group (48.8 ± 6.43) than the HC group (55.91 ± 0.3) (*p* = 0.007). The time required to complete the 5MWT was significantly higher for the TBI group (4.61 ± 1.18 s) compared to the HC (2.96 ± 0.58 s) group (*p* = 0.001) and similar group differences were observed for the 10MWT (*p* < 0.005) and TUG test (*p* < 0.005) (Table 3).

**FIGURE 4:**
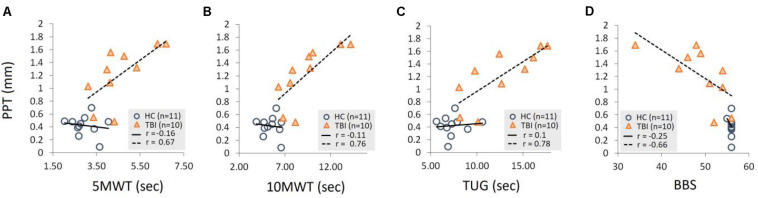
Demonstration of a linear relationship between the PPT (1 Hz) and time required to complete **(A)** 5MWT, **(B)** 10MWT, **(C)** TUG, and **(D)** scores for the BBS for the TBI group. No significant correlations were found for the HC group PPT data for all three frequencies. Also, no correlations were found for the 0.33 Hz and 0.5 Hz PPT data for the TBI group as reported in Table 4.

A Pearson product-moment correlation was run to determine the relationship between the PPT and functional measures of static and dynamic balance (Figure 4 and Table 4). For individuals with TBI, there was a significant positive correlation between the PPT (1 Hz) and the time required to complete 5MWT (*p* = 0.034), 10MWT (*p* = 0.012), and TUG (*p* = 0.008). For the TBI group, no significant correlation was found between 0.33 Hz PPT and time to complete 5MWT (*p* = 0.34), 10MWT (*p* = 0.13), and TUG (*p* = 0.29). Similarly, no significant correlation was found between 0.5 Hz PPT and 5MWT (*p* = 0.28), 10MWT (*p* = 0.18), and TUG (*p* = 0.14). For the HC group, no significant correlation was found between the PPT (all frequencies) and the time required to complete 5MWT, 10MWT, and TUG (see Table 4 and Figure 4). Furthermore, a significant negative correlation was found between the 1 Hz PPT and the BBS for the TBI group (*p* = 0.037), while no correlation was found for 0.33 Hz (*p* = 0.09) and 0.5 Hz PPT data (*p* = 0.17) (Table 4 and Figure 4). For the HC group, PPT data (all frequencies) showed no correlation with the BBS (Table 4 and Figure 4).

**TABLE 4:** Results of Pearson’s r correlation analysis between PPT and functional outcomes for both groups.

	Groups	PPT (0.33 Hz)	PPT (0.5 Hz)	PPT (1 Hz)
5MWT	HC (*n* = 11)	*r* = 0.19, *p* = 0.58	*r* = 0.07, *p* = 0.84	*r* = −0.16, *p* = 0.64
	TBI (*n* = 10)	*r* = 0.34, *p* = 0.34	*r* = 0.38, *p* = 0.28	*r* = 0.67, ***p* = 0.034**
10MWT	HC (*n* = 11)	*r* = 0.26, *p* = 0.44	*r* = 0.1, *p* = 0.77	*r* = −0.11, *p* = 0.75
	TBI (*n* = 10)	*r* = 0.51, *p* = 0.13	*r* = 0.46, *p* = 0.18	*r* = 0.76, ***p* = 0.012**
TUG	HC (*n* = 11)	*r* = 0.11, *p* = 0.75	*r* = 0.34, *p* = 0.31	*r* = 0.1, *p* = 0.76
	TBI (*n* = 10)	*r* = 0.38, *p* = 0.29	*r* = 0.5, *p* = 0.14	*r* = 0.78, ***p* = 0.008**
BBS	HC (*n* = 11)	*r* = −0.36, *p* = 0.28	*r* = −0.38, *p* = 0.25	*r* = −0.25, *p* = 0.46
	TBI (*n* = 10)	*r* = −0.57, *p* = 0.09	*r* = −0.48, *p* = 0.17	*r* = −0.66, ***p* = 0.037**

*Bold values represent statistically significant differences.*

## Discussion

The primary objective of this investigation was to quantify the sensory acuity to perturbations to the base of support during standing in individuals with TBI. Accurate perception of body-sway is critical in a dynamic setting to adapt to external stimuli and generate adequate motor responses for achieving balance control. Sensory acuity directly relates to perceptual mechanisms and impairment to sensory afferents as well as their integration after TBI could significantly contribute to impaired sensory acuity and balance dysfunction. Limited research specifically reports objective quantification of impairments to sensory integration after TBI (Peterka et al., 2018) and no research thus far has investigated sensory acuity in the individuals with TBI. This investigation presents an objective measure of sensory acuity in terms of PPT which goes beyond the biomechanical and functional markers of balance dysfunction and it is related to the process of sensory integration. The sensory organization test (SOT) has been widely used to assess contributions of visual and somatosensory inputs in maintaining balance during standing (Nashner and Peters, 1990). More recently, Peterka et al. proposed a novel central sensorimotor integration (CSMI) tests to quantify sensory integration by measuring the relative contributions of different sensory systems to balance control (Peterka et al., 2018). Though these tests provide an objective way to quantify sensory integration, the sensory acuity to external perturbations and its relation to balance function still remains to be studied in individuals with TBI. Further, sensory acuity in terms of detection threshold assessments to the whole-body stimuli have been reported (Fitzpatrick and McCloskey, 1994; Richerson et al., 2003, 2006; Puntkattalee et al., 2016), however limited data exists for individuals with TBI (Pilkar et al., 2016; Tanis et al., 2018). For the first time, a classical psychophysical approach was used to determine sensory acuity in terms of PPT in a sample of individuals with impaired balance. A lower PPT for a set of perturbations at a given frequency suggests a better perceptual ability to detect base of support perturbations during standing. The TBI group showed significantly elevated PPT values compared to the HC group for all three perturbation frequencies suggesting their diminished ability to perceive and report changes in their support surface during standing. Multi-sensory deficits are common due to brain lesions after TBI (Allison, 1999), and these deficits can lead to impaired sensory integration, reduced ability to use the optimal sensory system in different environmental contexts or over-reliance on a single sensory system, which is usually the visual system (Allison, 1999). However, Fitzpatrick and McCloskey (1994) showed that the visual thresholds for perceiving movement are higher than the proprioceptive thresholds at slower velocities of base of support movements in healthy individuals. In the context of PPT assessments, the perturbation frequencies are within the natural sway and amplitudes are kept small (<4 mm) by the algorithm as perturbations are confined by the sensory-threshold boundaries. Therefore, such perturbations may be difficult to perceive if only the visual system is used. As a result, sole reliance on the visual system while vestibular and somatosensory systems are impaired could significantly impact one’s ability to perceive the altered posture in relation to itself and the environment. In situations where multiple sensory modalities are available (e.g., PPT assessments), participants will yield thresholds that are equivalent to the sensory modality with the greatest acuity (Fitzpatrick and McCloskey, 1994). The vestibular system is only known to be engaged at much greater postural disturbances (Fitzpatrick and McCloskey, 1994) and visual system requires larger threshold amplitude (Fitzpatrick and McCloskey, 1994; Richerson et al., 2003). Hence, the majority of the contributions toward PPT where the perturbation frequencies and amplitudes are kept within the natural sway, could stem from the proprioceptive afferents. It is postulated that contributions from the tactile afferents to be minimal as all participants wore shoes on the platform and presence of footwear has shown to attenuate the tactile information compared to the barefoot condition (Robbins et al., 1995).

The PPT assessments were performed for three sets of perturbation frequencies. Similar to previously reported studies (Richerson et al., 2006), our selection of frequencies is based on the rationale that the our primary objective was to quantify the sensory acuity and not study the reactive postural strategies to the external stimuli. Therefore, frequencies ≤1 Hz kept the perturbations within the natural sway (Soames and Atha, 1982) which were appropriate for our assessments. Of the three perturbation frequencies, 1 Hz perturbations are curious based on two results −- (1) PPT for 1 Hz were significantly lower than 0.33 and 0.5 Hz with no significant difference between 0.33 and 0.5 Hz perturbations; and (2) a significant negative correlation was found between 1 Hz PPT and functional measures of balance (Figure 4) while no such relationship was observed for 0.33 and 0.5 Hz data for the TBI. These results may suggest that 0.33 and 0.5 Hz perturbations might not be differentiable by the sensory systems resulting in no significant perceptual differences for both groups. Further, these slow perturbations may not be sufficient enough to engage the sensory mechanisms that are relevant to influence the functional tasks hence showed no correlation with functional outcomes. On the other hand, 1 Hz perturbations could be sufficient enough to tap into impaired sensory mechanisms of TBI group (but still not large enough to tap into intact sensory system of the HC group) that are also relevant to functional balance tasks. This may have led to the linear relationships between the PPT at 1 Hz and functional measures suggesting that a lower PPT (i.e., the enhanced sensory acuity) could be critical for achieving adequate postural and functional control after TBI. For future investigations, 1 Hz may serve as guidance for selecting perturbation frequencies for similar experiments. Perturbations between 0.5 and 1 Hz could be explored to further confirm the dependency of sensory acuity on perturbation frequency as seen in Figure 2. The PPT assessments for perturbations faster than 1 Hz may induce additional postural strategies (hip) and may require extremely small and precise amplitudes to reach to threshold detection, however such perturbations may not be practically deliverable using the existing Neurocom CRS system or in fact, may not yield the PPT.

Enhanced perception (i.e., lower PPT) requires integration and interpretation of the multi-sensory afferents as well as the capability to handle attentional demands. Therefore, in addition to impaired sensory integration, an elevated PPT could also stem from the deficits in attention that occur in 39–62% of TBI survivors (Marsh et al., 2016). Selective attention is essential for dynamic aspects of activities of daily living (Straudi et al., 2017) and individuals with balance impairments due to deficits in their automatic postural responses (APRs) (Woollacott and Shumway-Cook, 2002) rely more heavily on attentional mechanisms during standing. Attentional deficits post TBI and potentially impaired APRs due to impaired sensory integration could interfere with a TBI survivor’s ability to safely complete motor tasks (Ponsford and Kinsella, 1992). Our novel PPT outcome therefore not only reflects the perceptual and attentional indicators of balance deficit but also presents a potentially quantifiable link between the sensory acuity and functional tasks. The absence of significant correlations between the PPT and functional measures for the HC group could potentially imply less reliance on attentional mechanisms and more on their unaffected APRs (Puntkattalee et al., 2016) as well as intact attentional mechanisms.

The literature on TBI balance suggests that the level and characteristics of balance impairments are related to the severity and location of the brain damage (Allison, 1999). The PPT outcome reported in this investigation as a measure of sensory acuity is a manifestation of cognitive (attention) and sensory components. Therefore, the results reported could be influenced by injury characteristics such as time since injury (TSI), severity, location of lesions, etc. Injury characteristics that directly affect cognition (attention), sensory and motor components are expected to show impaired sensory acuity (elevated PPT values). It is expected that the individuals with TBI in the acute stage with the severe symptoms will most likely show elevated PPT values and with the recovery of sensorimotor function over time due to neuroplasticity or rehabilitation, PPT would decrease. Moreover, the individuals with damages to the spinocerebellar tract and the anterior lobe of the cerebellum could show elevated values of PPT as legions to these areas are known to affect the transmission and perception of somatosensation needed to detect the location of body segments in relation to each other and the location of the body in relation to the base of support (Allison, 1999).

### Limitations and Future Considerations

The limitations of the current work are its small sample size and heterogeneity within the TBI group in terms of the severity of the injury as well as sex. Heterogeneity within the population is a common challenge in characterizing the balance after TBI due to the complexity of injury and deficits (Allison, 1999). A larger homogeneous sample of TBI (based on the severity of injury, TSI, legions, and functional capability) with equal distribution of male and female participants needs to be assessed at multiple time-points to comprehensively understand the PPT as an outcome measure. Furthermore, the current unidirectional (applying perturbations only in AP direction) approach of the posturography assessment limits the understanding of the role sensory acuity plays in maintaining balance. It has been suggested that the keys to improving balance after a TBI include training methods that are specific and require multiple adaptive responses (Horak et al., 1997; Huang et al., 2006), and as a result, a multidirectional approach for perturbation-based assessment and training is recommended. Finally, the presented method to evaluate sensory acuity employs multi-sensory approach which may not be able to isolate the impairments specific to individual sensory system. However, this investigation focuses on objective evaluation of sensory acuity and its potential connection to the balance dysfunction after TBI. Therefore, use of multi-sensory approach is applicable as most of the functional balance tasks employ a multi-sensory approach. The PPT outcome presented in this investigation can serve as an additional marker of balance dyfunction in addition to the functional and biomechanical (CoP, CoM) outcomes after TBI. The interventions that specifically target the sensory mechanisms have shown to be effective in improving standing balance. E.g., Charkhkar et al. (2020) showed that the enhanced perception of the plantar pressures under the prosthetic feet achieved using artificial sensory feedback can significantly improve the postural stability of lower limb amputees. Similarly, Petrini et al. (2019) showed that real-time tactile and proprioceptive feedback provided by sensory neuroprosthetic promoted improved mobility, fall prevention, and agility during active tasks in transfemoral (above-knee) amutees. These investigations show that the manipulation and augmentation of sensory feedback is critical to enhance balance and mobility. Our novel outcome, PPT, can be integrated into balance training paradigms to provide perturbations that engage and enhance proprioception and somatosensation and improve balance after TBI.

## Conclusion

The current work presented the PPT as a new metric for the objective assessment of the sensory acuity to perceive external horizontal perturbations to the base of support during standing in individuals with a TBI. The TBI group showed significantly elevated PPTs compared to the HC group, suggesting their diminished ability to perceive changes to perturbation-induced sway. A significant correlation between the PPTs and functional outcomes was found for the TBI group, demonstrating the critical role perceptual ability may play in achieving improved balance function after injury. Therefore, sub-threshold perturbations that engage perceptual mechanisms could be important to include along with the supra-threshold perturbations that engage the compensatory mechanisms during balance rehabilitation after TBI.

## Data Availability Statement

The datasets generated for this study are subject to the following licenses/restrictions: Data availability will be subjected to the funding agency guidelines. Requests to access these datasets should be directed to https://www.state.nj.us/health/njcbir/.

## Ethics Statement

The studies involving human participants were reviewed and approved by the Kessler Foundation Institutional Review Board. The patients/participants provided their written informed consent to participate in this study.

## Author Contributions

RP and KN designed the study. NE and OI performed the data collection. RP, KK, and AV performed the data analysis and prepared the manuscript. KK and RP performed the statistical analysis. All authors contributed to the article and approved the submitted version.

## Conflict of Interest

The authors declare that the research was conducted in the absence of any commercial or financial relationships that could be construed as a potential conflict of interest.
